# Environmental and meteorological factors linked to malaria transmission around large dams at three ecological settings in Ethiopia

**DOI:** 10.1186/s12936-019-2689-y

**Published:** 2019-02-26

**Authors:** Solomon Kibret, G. Glenn Wilson, Darren Ryder, Habte Tekie, Beyene Petros

**Affiliations:** 1 Ecosystem Management, School of Environmental and Rural Science, University of New England, Armidale, NSW 2351 Australia; 2 Present Address: Program in Public Health, University of California, Irvine, CA 92697 USA; 3 Present Address: Department of Biology, University of Southern Denmark, Campusvej 55, 5230 Odense M, Denmark; 4 Department of Zoological Sciences, College of Natural Sciences, Addis Ababa University, Addis Ababa, Ethiopia; 5 Department of Microbial, Cellular and Molecular Biology, College of Natural Sciences, Addis Ababa University, Addis Ababa, Ethiopia

**Keywords:** Malaria, Climate, Environment, Reservoir shoreline, Water level, Dam, Ethiopia

## Abstract

**Background:**

A growing body of evidence suggests that dams intensify malaria transmission in sub-Saharan Africa. However, the environmental characteristics underpinning patterns in malaria transmission around dams are poorly understood. This study investigated local-scale environmental and meteorological variables linked to malaria transmission around three large dams in Ethiopia.

**Methods:**

Monthly malaria incidence data (2010–2014) were collected from health centres around three dams located at lowland, midland and highland elevations in Ethiopia. Environmental (elevation, distance from the reservoir shoreline, Normalized Difference Vegetation Index (NDVI), monthly reservoir water level, monthly changes in water level) and meteorological (precipitation, and minimum and maximum air temperature) data were analysed to determine their relationship with monthly malaria transmission at each dam using correlation and stepwise multiple regression analysis.

**Results:**

Village distance to reservoir shoreline (lagged by 1 and 2 months) and monthly change in water level (lagged by 1 month) were significantly correlated with malaria incidence at all three dams, while NDVI (lagged by 1 and 2 months) and monthly reservoir water level (lagged by 2 months) were found to have a significant influence at only the lowland and midland dams. Precipitation (lagged by 1 and 2 months) was also significantly associated with malaria incidence, but only at the lowland dam, while minimum and maximum air temperatures (lagged by 1 and 2 months) were important factors at only the highland dam.

**Conclusion:**

This study confirmed that reservoir-associated factors (distance from reservoir shoreline, monthly average reservoir water level, monthly water level change) were important predictors of increased malaria incidence in villages around Ethiopian dams in all elevation settings. Reservoir water level management should be considered as an additional malaria vector control tool to help manage malaria transmission around dams.

**Electronic supplementary material:**

The online version of this article (10.1186/s12936-019-2689-y) contains supplementary material, which is available to authorized users.

## Background

Malaria is a serious public health challenge in sub-Saharan Africa, with an estimated 200 million cases of malaria in 2017 alone [1]. This region accounts for 92% of the global malaria burden [1]. A number of environmental, climatic, seasonal, and ecological factors determine the occurrence and intensity of malaria transmission. For instance, while rainfall limits the availability of breeding habitats for mosquito vectors, temperature determines the length of mosquito larvae development and the rate of growth of the malaria parasites inside the vector [2, 3]. In addition, environmental modifications, such as the construction of dams and irrigation schemes, also affect the type and distribution of mosquito breeding habitats [4, 5].

In Africa, dams have been demonstrated to enhance rates of malaria transmission in areas of unstable transmission [5, 6]. Increased malaria incidence following dam construction was reported around several African dams [7–14]. Overall, dams have been shown to contribute to over 1 million malaria cases annually in sub-Saharan Africa [15]. However, the extent to which various environmental and climatic factors may have contributed to enhanced rates of malaria transmission around these sites remains poorly understood.

Climatic variables such as precipitation and air temperature are important determinants of the spatial distribution and relative abundance of malaria vector species in Africa [16]. For instance, in Africa, *Anopheles gambiae* is the predominant species in high rainfall environments, while *Anopheles arabiensis* is more common in arid areas [17, 18]. However, climatic conditions are also inter-related with elevation. For example, air temperature decreases as elevation increases, and consequently the abundance and species composition of malaria vectors may change significantly with elevation [16].

In Ethiopia, local increases in malaria rates have been blamed on the establishment of new dams [9, 10, 12–14]. A new era of dam construction currently underway in Ethiopia [19] has also elevated concerns for the public health impact of these infrastructures. Yet, dams are important contributors to Ethiopia’s economic development and food security. However, a poor understanding of their effects on malaria transmission in different ecological settings represents a critical barrier to the sustainability of water storage infrastructures.

Understanding how different environmental and climatic factors affect rates of malaria transmission is required to develop appropriate disease control tools. A recent review suggested that the relationship between dams and malaria incidence varies across ecological settings [15]. However, the study did not investigate how environmental (other than the presence of dams) and climatic factors vary across these ecological settings and, in turn, affect rates of malaria incidence around dams. The present study aims to investigate relationships among a number of environmental and meteorological factors associated with malaria transmission around Ethiopian dams in three ecological settings: highland, midland and lowland elevations.

## Methods

**Fig. 1:**
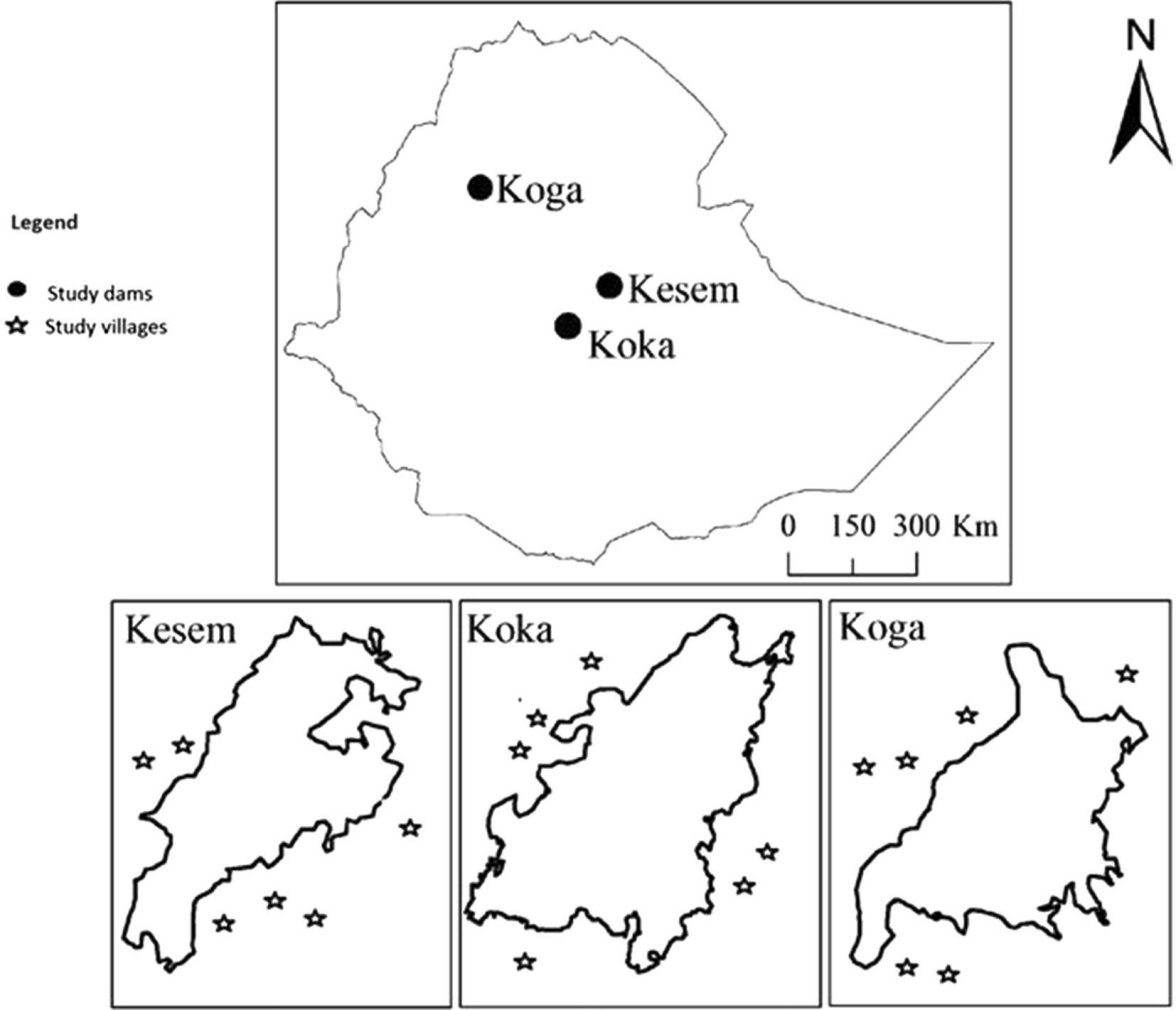
Map of the study area and location of study villages in relation to the reservoir shorelines

The present study was conducted around three dams in Ethiopia: Kesem Dam [975 m above sea level (asl)], Koka Dam (1551 m asl) and Koga Dam (1980 m asl) (Fig. 1). At each dam, six villages within a 5-km radius of the reservoir shoreline were randomly selected for this study. Only villages located upstream of the dam were included to avoid potentially confounding influences of the downstream river environment. Time series malaria case data as well as environmental and meteorological data were analysed to determine factors linked to malaria transmission at each dam setting.

Kesem Dam (hereafter referred as the lowland dam) is located on the Awash River in the lowlands of the Ethiopian Rift Valley, 225 km east of Addis Ababa, the capital of Ethiopia. Its crest height of 25 m stores a maximum of 500 million cu m of water, covering an area of 200 sq km. The maximum length of the shoreline at full capacity is 55.4 km. The primary purpose of the dam is to irrigate 20,000 ha of land for sugarcane production downstream. The area is characterized as semi-arid with a mean daily temperature of 27 °C. The hottest month is May (mean daily temperature is 38 °C) and the coldest is December (average daily temperature is 18 °C). The area receives an average annual total rainfall of 600 mm; the main rainy season (June to August) accounts for 80% of the total rainfall. An estimated population of 35,000 lives within a 5-km radius of Kesem reservoir [20].

Koka Dam (hereafter referred as the midland dam) is located in the Ethiopian Rift Valley in Central Ethiopia, 100 km south of Addis Ababa. It has a crest height of 42 m, and a full water storage capacity of 1188 million m^3^. The surface area of the reservoir at full capacity is 236 sq km and the length of the reservoir at full storage capacity is 86 km. The primary purpose of the dam is to generate 43.2 MW of electricity from three turbines (approximately 6% of the current total grid-based generating capacity of the country). Currently, the Wonji sugarcane irrigation scheme (6000 ha), located approximately 12 km downstream of the dam, is also dependent on releases from Koka Dam. In addition, the dam is used for flood control. The area receives a total annual rainfall of 850 mm and the mean daily temperature is 22 °C (National Meteorological Agency, unpublished report). The hottest month is May (mean daily temperature is 29 °C) and the coldest is December (average daily temperature is 12 °C). An estimated population of 29,000 lives within 5 km of the reservoir [20].

Koga Dam (hereafter referred as the highland dam) is located on the Koga River, one of the major tributaries of the Blue Nile River, 560 km northwest of Addis Ababa. The dam has a storage capacity of 83.1 million m^3^ and surface area of 175 sq km. It was commissioned in 2009 to irrigate 7000 ha of wheat, corn and teff crops. The rainy season (June to August) generates about 70% of the run-off feeding the Koga River [21]. The length of the reservoir shoreline at full capacity is 120 km. The area is characterized as highland with a mean daily temperature of 19 °C and receives an average annual rainfall of 1500 mm. The hottest month is May (average daily temperature is 26 °C) and the coldest is January (average daily temperature is 10 °C). An estimated 32,680 people live within 5 km of the reservoir [20].

Malaria is the leading public health challenge in all study villages, peaking between September and November following the months of the rainy season (June to August). The inhabitants of the study villages are agrarians, and cattle herding is also common. *Plasmodium falciparum* is the predominant malaria-causing parasite, accounting for 70–80% of malaria infections (Oromia Health Bureau, unpublished report). The remaining malaria infections are due to *Plasmodium vivax. Anopheles arabiensis* is the major malaria vector species in the study area while *Anopheles pharoensis* plays a secondary role [12]. Potential mosquito breeding habitats in the study area include shoreline puddles, irrigation canals, rain pools, and man-made pools [22].

### Retrospective malaria case data

Five years (January 2010 to December 2014) of weekly malaria data were obtained from the health facilities at each of the dam sites. Inhabitants of the study area visit these health facilities to receive medical attention. At each health centre, each febrile case was tested by a trained laboratory technician for malaria using microscopic blood screening to distinguish between *P. falciparum* and *P. vivax*. Test results were recorded in the laboratory registry, along with data of outpatient name, age, gender, and residency. These data were de-identified and exported to Microsoft Excel and SPSS for analysis. These data were checked for completeness and correctness by cross-referencing the laboratory registry with the outpatient registry at each clinic. The completeness of the registry ranges from 75 to 82% across the health facilities. A recent external quality assessment of the skills of the microscopists in these health facilities identified that 80% of slides were correctly recorded with correct parasite quantification [23]. For quality control, five positive and five negative slides were randomly selected from health facilities each month, and taken to the District Laboratory for re-checking.

### Environmental data

The environmental data used for this study comprised village elevation, village distance from reservoir shoreline, Normalized Difference Vegetation Index (NDVI) and reservoir water level. Village elevation was recorded using a handheld Geographical Positioning System receiver (GPSMAP 60CSx, Garmin International Inc., USA). For each study village, data on monthly distance from reservoir shoreline were acquired from the European Space Agency image repository [24]. These images had a resolution of 150 × 150 m, were geo-referenced, and taken in the first week of each month between January 2010 and December 2014. These were then imported to ArcGIS 9.2 to estimate the distance between the centre of each study village and the nearest reservoir shoreline for each month of the study period.

Monthly NDVI data for the study villages were acquired from the US National Oceanic and Atmospheric Administration (NOAA) that documents data of the Moderate Resolution Imaging Spectroradiometer (MODIS) instruments on-board the Terra and Aqua Satellites. These satellites provide a vegetation survey at a 250-m spatial resolution every 16 days [25]. The MODIS NDVI products are computed from atmospherically corrected, bi-directional surface reflectances that have been masked for water, clouds, heavy aerosols, and cloud shadows. NDVI is a measure of vegetation condition, used here as a proxy for mosquito habitat availability [26]. NDVI values vary between + 1.00 and − 1.00; the higher the NDVI value, the denser the green vegetation.

Daily reservoir water level data were obtained for each dam from the Ethiopian Electricity and Power Corporation, and the Ministry of Water Resources for the duration of the study period (January 2010 to December 2014). These were then exported to Microsoft Excel and SPSS for analysis. The data were aggregated to monthly averages and monthly changes in water level (i.e., calculated by subtracting the amount of the reservoir water level (m) at the end of a month from that at the beginning of the month; negative values indicate receding water level while positive values indicate increasing water levels) for each of the three dams. The objective of including monthly changes in water level was to determine how the magnitude of change in water level correlates with malaria incidence as it directly affects the nature of the shoreline for mosquito breeding habitats.

### Meteorological data

Five years (January 2010 to December 2014) of daily meteorological data, including total rainfall (mm), and mean daily minimum and maximum air temperature (°C), were obtained from three meteorological stations at each of the three study dam sites. Any missing values were replaced with daily average data from the closest neighbouring station. Data were then aggregated to monthly averages and exported to Microsoft Excel and SPSS for analysis.

### Statistical analysis

To satisfy the assumptions of individual statistical analyses, first the normality in the distribution of monthly malaria incidence, environmental and meteorological data sets was tested using SPSS. Temperatures (both minimum and maximum), reservoir water level (and change in water level) and NDVI values were normally distributed and analysed as explanatory variables. Malaria incidence (dependent variable) and precipitation data were found to have a skewed distribution and thus were log-transformed accordingly.

For each village, malaria incidence was calculated as the number of cases per 1000 population. One-way Analysis of Variance (ANOVA), followed by a Tukey’s test, was used to test for the differences in malaria incidence between the three dam sites.

Average monthly meteorological data (precipitation, minimum and maximum air temperature) were calculated and lagged by one and 2 months to allow time for mosquitoes and malaria parasites to complete their life cycle prior to the expression of any malaria incidence. Similarly, monthly NDVI data were also lagged by one and 2 months to allow time for mosquito development. Monthly relative humidity data were not included in the analysis due to there being too many missing values for the duration of the study period. To determine any correlation between meteorological/environmental variables and malaria incidence at each dam site, univariate associations were first examined by regressing single explanatory factors (i.e., environmental and meteorological variables) against malaria incidences for each dam site. Since there might be cross-correlation between independent variables over time, cross-correlation analyses were conducted. When the correlation coefficient for the association between the independent variables was greater than 0.5, these variables were analysed in Autoregressive Integrated Moving Averages (ARIMA) to avoid multicollinearity [27]. After the effect of any auto-correlation had been removed by the ARIMA procedure, stepwise forward multiple regression analyses were used to identify the meteorological/environmental factors that best explained malaria incidence at each dam site. Only those variables with a significant correlation (*P* < 0.05) with malaria incidence were added in the multiple regression models. Among lagged variables, only those with the highest correlation (r^2^ > 0.5) were included to these analyses. All analyses were performed using Microsoft Excel and SPSS Version 21 software.

## Results

### Spatial and temporal variation in malaria incidence

**Fig. 2:**
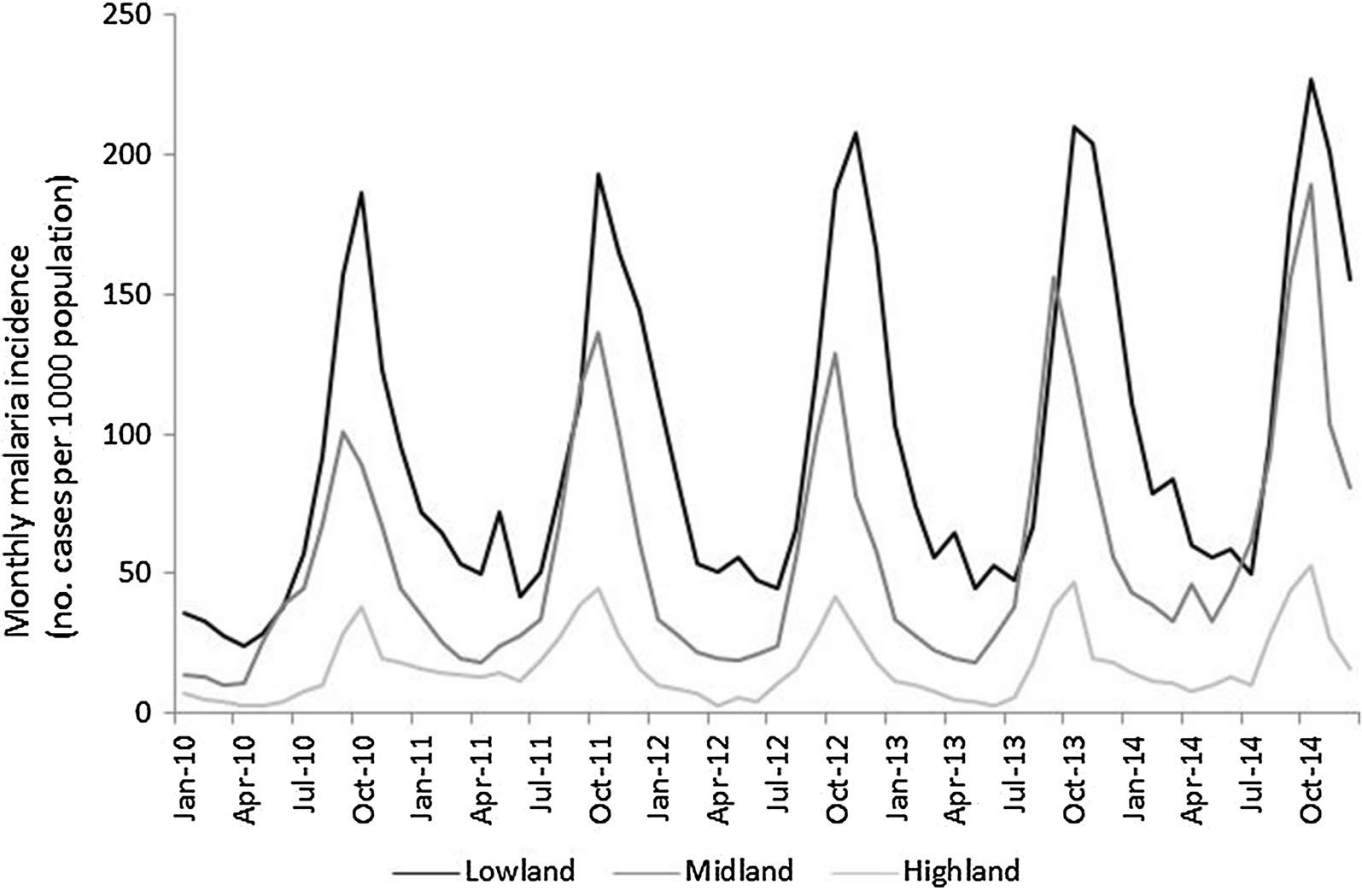
Temporal variation in monthly malaria incidence in reservoir communities at the lowland, midland and highland dams in Ethiopia, 2010–2014

**Fig. 3:**
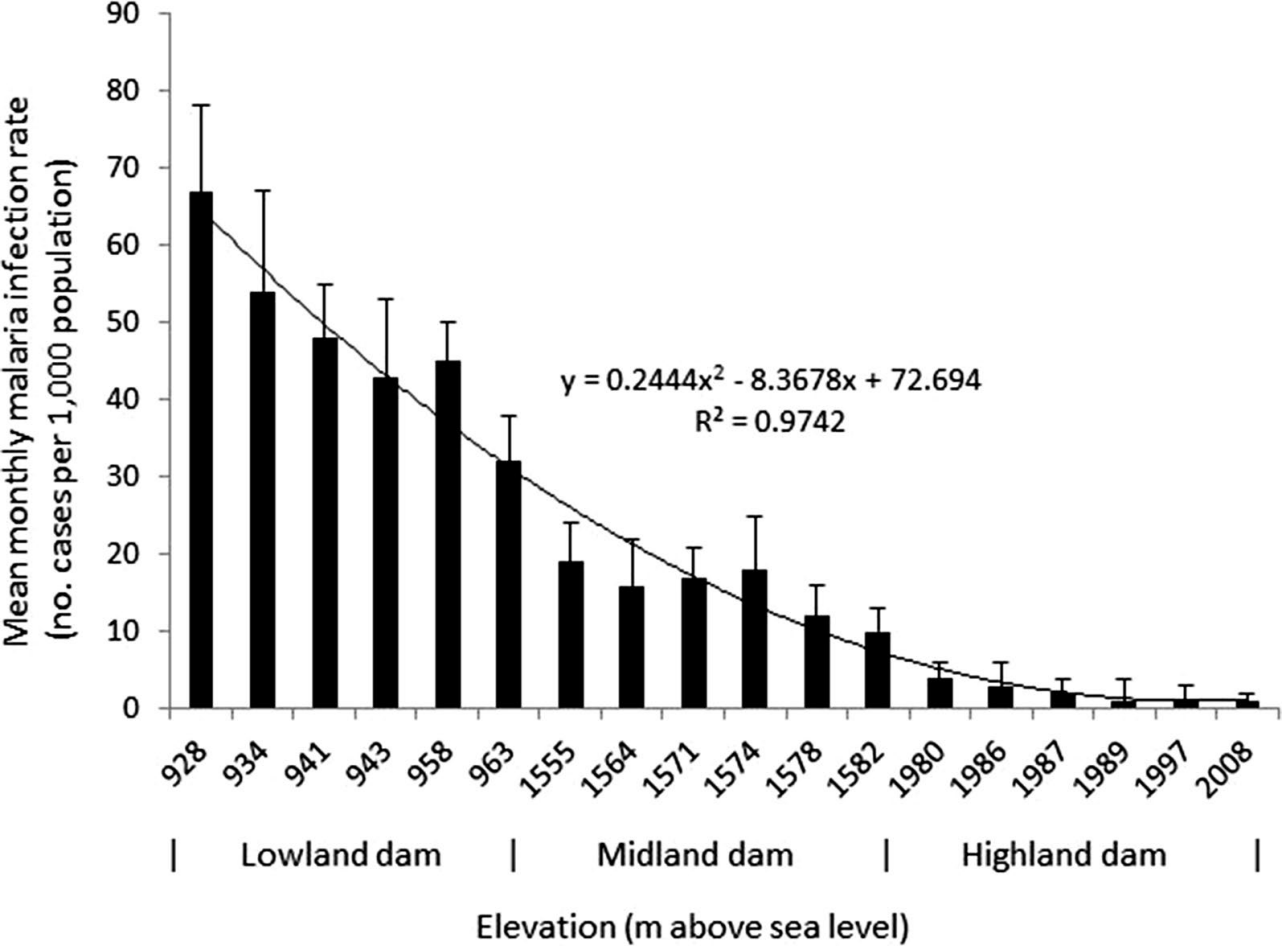
Relationship between malaria incidence and village elevation at the lowland, midland and highland dams in Ethiopia

Mean monthly malaria incidence was 1.7- and 5.6-times higher at the lowland dam (mean = 96.3; 95% CI = 81.5–111.0; ANOVA: F = 54.7; *P* < 0.001) than the midland (mean = 56.7; 95% CI = 45.9–67.4) and highland dam (mean = 17.2; 95% CI = 13.9–20.4) dams, respectively (Table 1). The temporal variation in malaria incidence at the three dams showed a seasonal peak between September and November at all study dams (Fig. 2). Differences in malaria incidence between villages and years at each dam site, however, were not statistically significant (ANOVA, *P *> 0.05). Malaria incidence was generally strongly correlated with elevation (r^2^ = 0.97; *P* < 0.05): malaria incidence decreased as elevation increased (Fig. 3).

**Table 1:** Summary of monthly mean malaria incidence at the three study dams in Ethiopia, 2010–2014

Dam location	Mean malaria incidence	95% CI	Odds ratio	P*
Lowland	96.3	81.5–111.0	1	–
Midland	56.7	45.9–67.4	1.7	< 0.01
Highland	17.2	13.9–20.4	5.6	< 0.01

* ANOVA test. The difference in malaria incidence between midland and highland dam was also significant (Tukey test, *P* < 0.01)

### Impact of environmental factors on malaria incidence

**Fig. 4:**
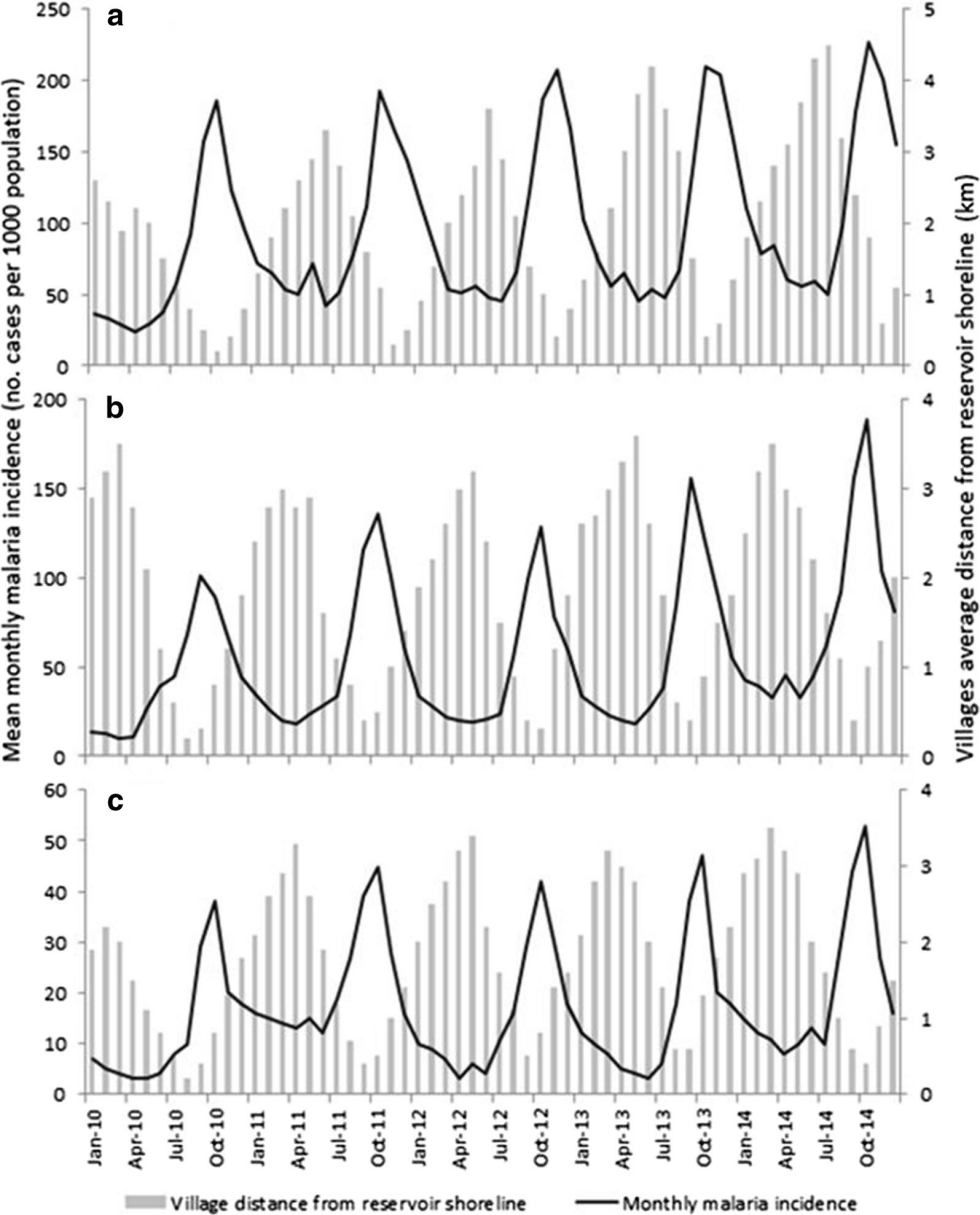
Temporal variation in monthly malaria incidence and villages distance from reservoir shoreline at **a** lowland, **b** midland and **c** highland dams in Ethiopia. NB: Y-axis scales vary between the three plots

Village proximity to a reservoir shoreline was negatively correlated with malaria incidence in all the three dam sites: the shorter a village’s distance to the shoreline, the higher the malaria incidence in the following month (Fig. 4). Indeed, approximately 69% (annual average from 51 to 86%) of annual malaria cases occurred when a village’s distance was less than 2 km from the shoreline. This trend was consistent across the three dam sites.

**Fig. 5:**
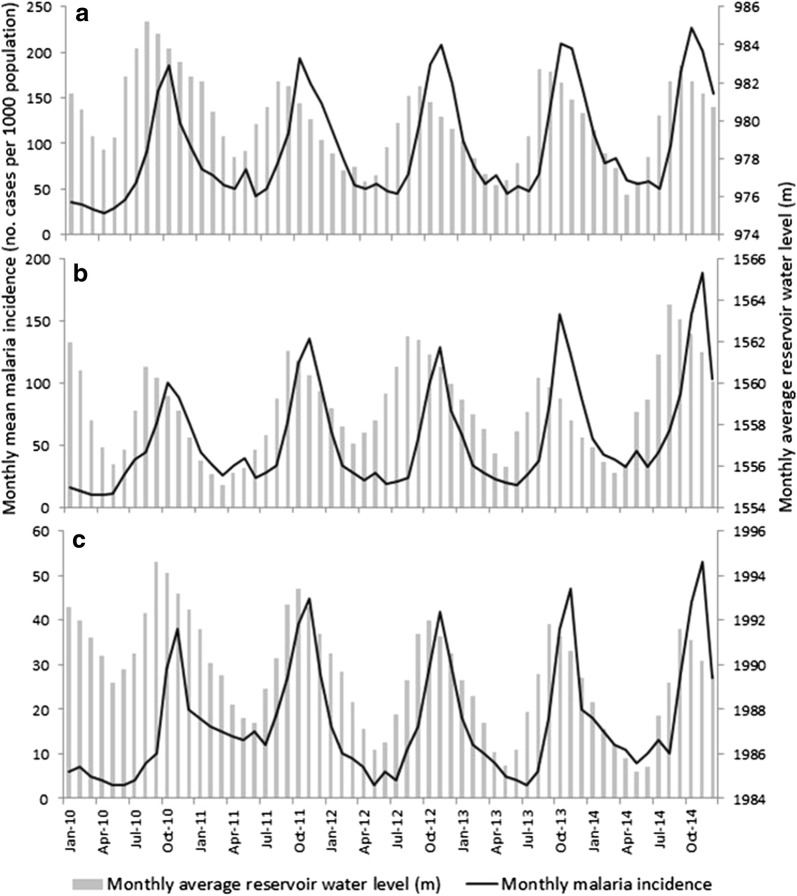
Temporal variation in monthly malaria incidence and monthly average reservoir water level at **a** the lowland, **b** midland and **c** highland study dams in Ethiopia, 2010–2014. NB: Y-axis scales vary between the three plots

**Fig. 6:**
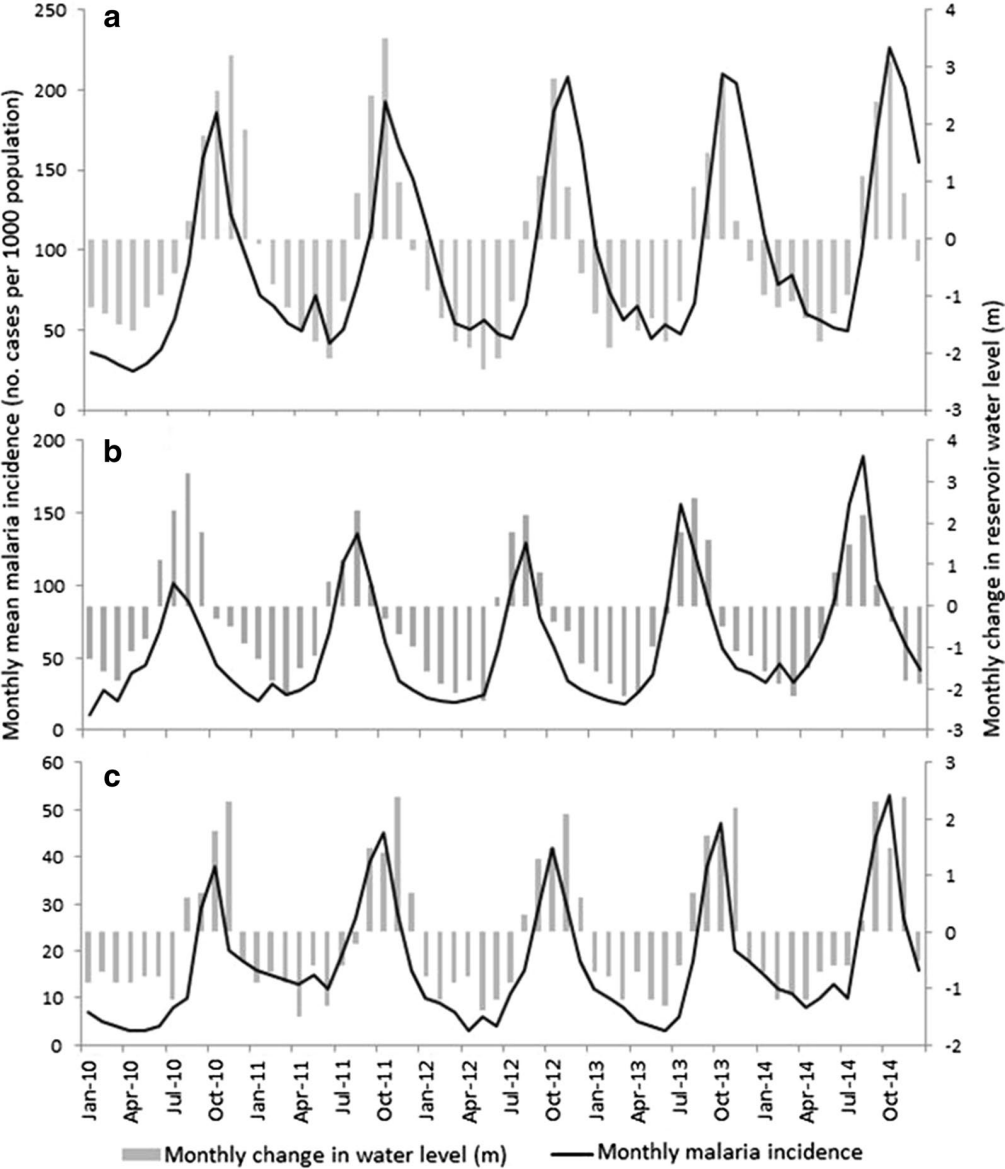
Temporal variation in monthly malaria incidence and monthly change in reservoir water level at the **a** lowland, **b** midland and **c** highland study dams, Ethiopia. NB: Y-axis scales vary between the three plots. Negative water level changes refer to receding water levels

**Fig. 7:**
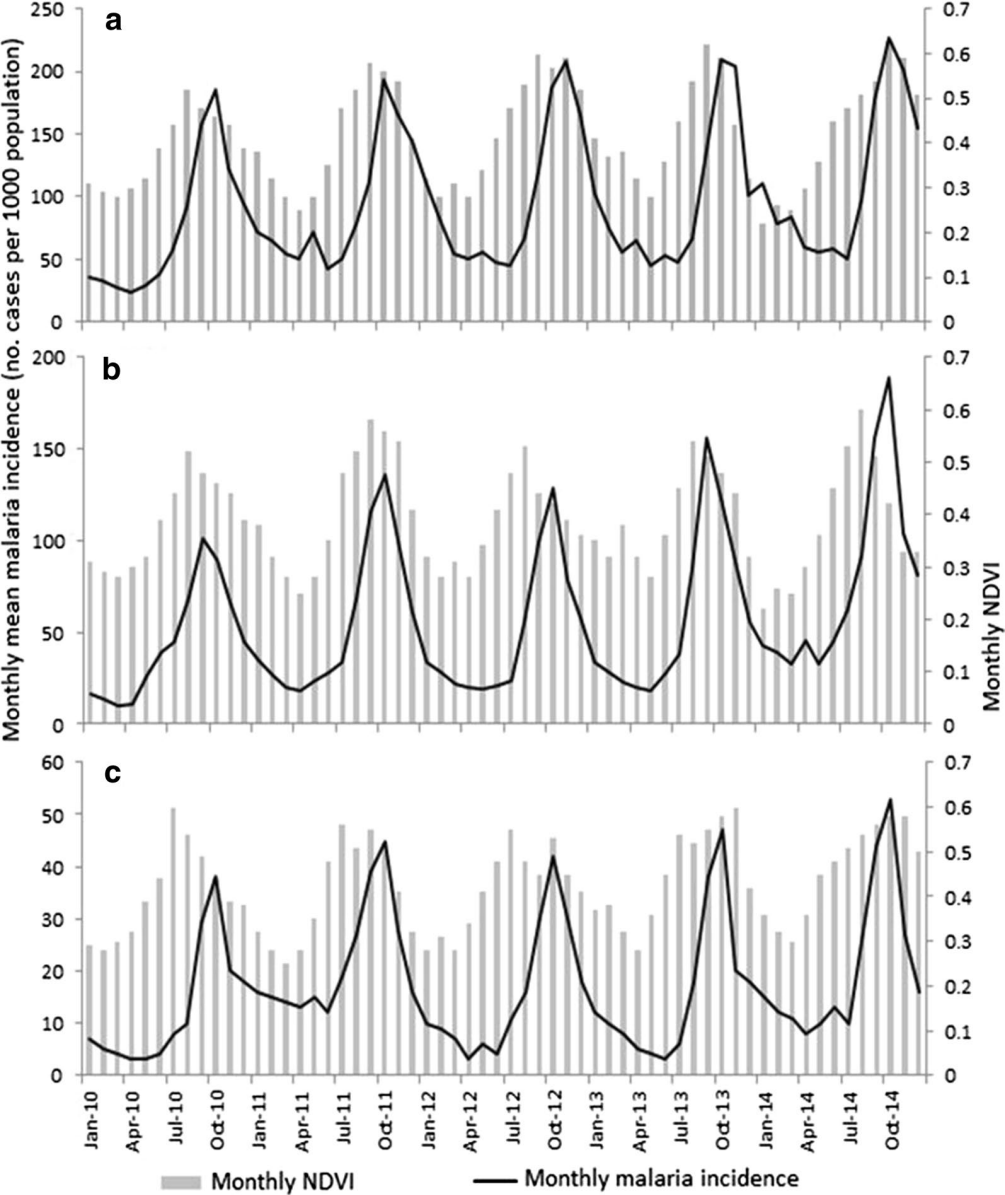
Relationship between monthly malaria incidence and monthly NDVI at **a** lowland, **b** midland and **c** highland dams in Ethiopia. NB: Y-axis scales vary between the three plots

Malaria incidence peaked following the months of high reservoir water level (Fig. 5). There was generally a 2-month lag-time between peak water level and peak malaria incidence, which was consistent across the dams. Similarly, malaria peaks also followed peaks in positive water level change at each dam (Fig. 6), with a lag-time ranging from 1 month (highland dam) to 2–3 months (midland and lowland dams). Likewise, high NDVI levels were associated with peaks in malaria incidence either 1–2 (lowland and midland dams) or 3 months (highland dam) later (Fig. 7).

Univariate analysis detected significant relationships between environmental variables and malaria incidence across the three dams (Table 2). NDVI (lagged by 1 and 2 months; r = 0.567 and 0.669, respectively), village distance from the reservoir shoreline (lagged by 1 and 2 months; r = − 0.598 and − 0.441, respectively), monthly average reservoir water level (lagged by 2 months; r = 0.362) and monthly change in reservoir water level (lagged by 1 month; r = − 0.616) were significantly associated with monthly malaria incidence at the lowland dam. At the midland dam, distance from reservoir shoreline (lagged by 1 and 2 months; r = − 0.455 and − 0.368, respectively), NDVI (lagged by 2 months; r = 0.452), monthly average reservoir water level (lagged by 2 month; r = 0.408) and monthly change in reservoir water level (lagged by 1 and 2 months; r = − 0.481 and − 0.366, respectively) were significantly associated with malaria incidence. At the highland dam, a strong correlation was found between monthly malaria incidence and distance from reservoir shoreline (lagged by 1 and 2 months; r = 0.487 and − 0.377, respectively) and monthly changes in reservoir water level (lagged by 1 month; r = − 0.301).

**Table 2:** Correlation between environmental variables and monthly malaria incidence at the lowland, midland and highland dams in Ethiopia

Environmental variable	Pearson’s correlation with mean monthly malaria incidence		
	Lowland dam	Midland dam	Highland dam
NDVI	0.341	0.255	0.127
NDVI lagged by 1 month	0.567*	0.303	0.239
NDVI lagged by 2 months	0.669*	0.452*	0.302
Village distance from reservoir shoreline	− 0.252	− 0.132	− 0.103
Village distance from reservoir shoreline lagged by 1 month	− 0.598*	− 0.455*	− 0.487*
Village distance from reservoir shoreline lagged by 2 months	− 0.441*	− 0.368*	− 0.377*
Monthly average reservoir water level	0.231	0.312	0.121
Monthly average reservoir water level lagged by 1 month	0.296	0.324	0.209
Monthly average reservoir water level lagged by 2 months	0.362*	0.408*	0.299
Monthly change in reservoir water level	− 0.124	− 0.235	− 0.191
Monthly change in reservoir water level lagged by 1 month	− 0.616*	− 0.481*	− 0.694*
Monthly change in reservoir water level lagged by 2 months	− 0.244	− 0.366*	− 0.301

* Significant correlation at *P *< 0.05

### Impact of meteorological variables on malaria incidence

**Fig. 8:**
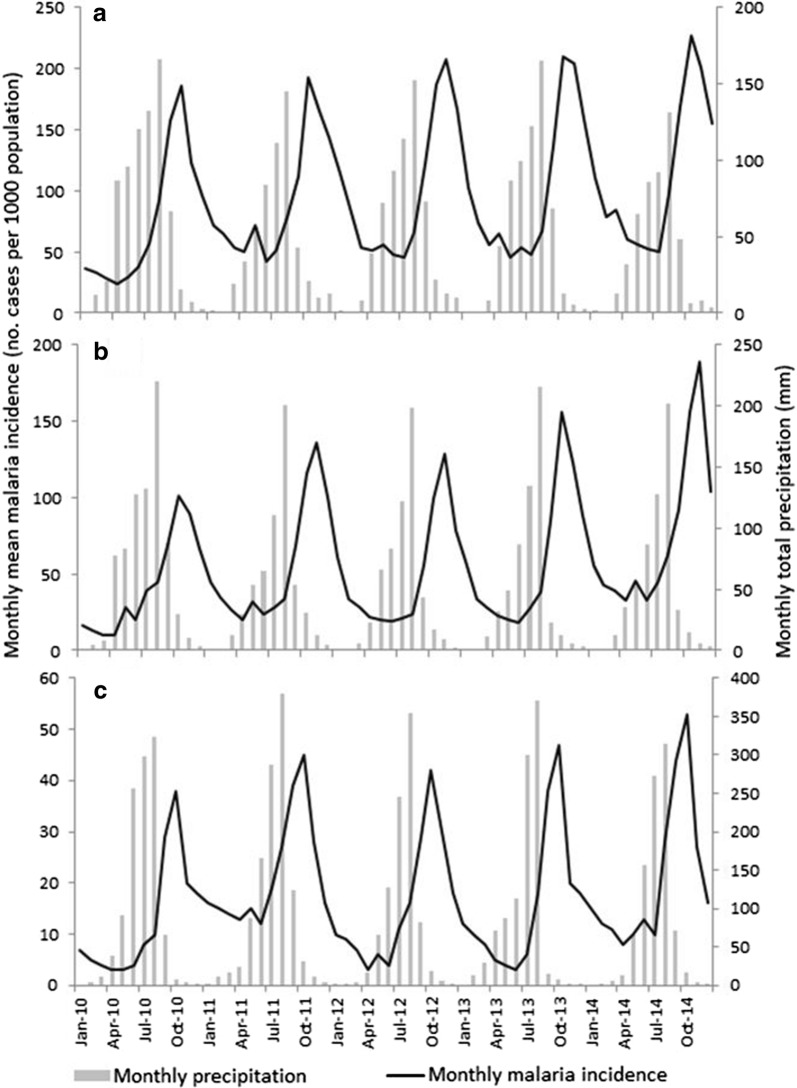
Temporal variation in monthly malaria incidence and monthly total precipitation at the **a** lowland, **b** midland and **c** highland study dams, Ethiopia. NB: Y-axis scales vary between the three plots

**Fig. 9:**
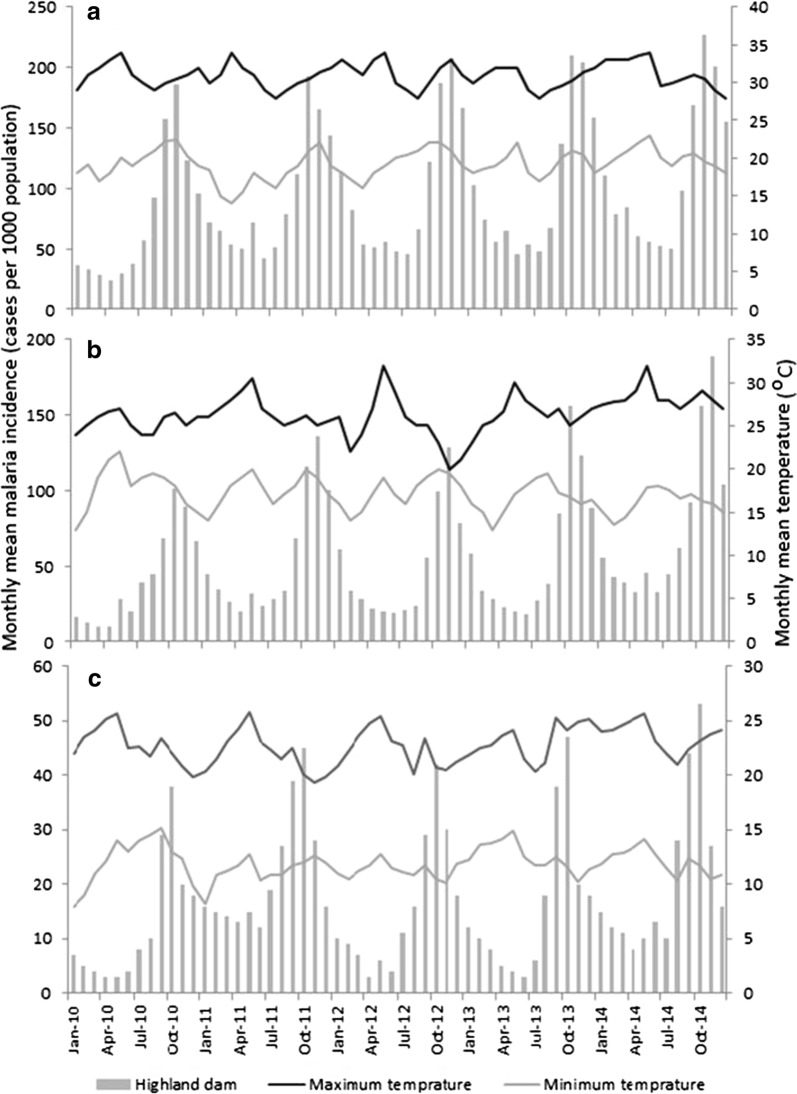
Temporal variation in malaria incidence and minimum and maximum air temperatures at the **a** lowland, **b** midland and **c** highland study dams, Ethiopia. NB: Y-axis scales vary between the three plots

A peak in malaria incidence followed mid-year peaks in rainfall at each of the three dams (Fig. 8). Lag times were relatively consistent between dams, ranging from an average of 2.4 or 2.6 months at the lowland and midland dams to 2.0 months at the highland dam. A peak in malaria incidence tended to occur a month following peaks in minimum air temperature at the lowland and highland dams, but from 1 to 2 months following the same peaks at the midland dam (Fig. 9). However, the relationship between seasonal malaria incidence and variation in maximum air temperature was less clear. At the lowland dam, malaria peaks occurred 2 to 4 months following late-summer troughs in maximum air temperature, but from 0 to 3 months following similar troughs at the midland dam. The same pattern was less consistent at the highland dam, with malaria incidence peaks tending to follow minor September peaks in maximum air temperature.

Univariate analysis of the influence of meteorological variables on seasonal malaria incidence indicated differences in variables that were significantly associated with malaria incidence between the three dam sites (Table 3). At the lowland dam, monthly total precipitation lagged by 1 and 2 months (r = 0.414; r = 0.672, respectively) were the only variables with a significant correlation with monthly malaria incidence. At the midland dam, monthly total precipitation lagged by 2 months (r = 0.329) and monthly mean minimum temperature lagged by 1 and 2 months (r = 0.501; r = 0.612, respectively) were significantly correlated with monthly malaria incidence. At the highland dam, monthly mean minimum (r = 0.419; r = 0634) and maximum (r = 0.364; r = 0.451) air temperature lagged by 1 and 2 months were significantly correlated with monthly malaria incidence.

**Table 3:** Correlation between climatic variables and monthly malaria incidence at the lowland, midland and highland dams in Ethiopia

Climate variable	Pearson’s correlation with mean monthly malaria incidence		
	Lowland dam	Midland dam	Highland dam
Monthly total precipitation	0.213	0.107	0.042
Monthly total precipitation lagged by 1 month	0.414*	0.268	0.211
Monthly total precipitation by 2 months	0.672*	0.376*	0.282
Monthly mean minimum temperature	0.231	0.184	0.302
Monthly mean minimum temperature lagged by 1 month	0.195	0.342*	0.419*
Monthly mean minimum temperature lagged by 2 months	0.232	0.399*	0.634*
Monthly mean maximum temperature	0.161	0.103	0.147
Monthly mean maximum temperature lagged by 1 month	0.208	0.198	0.364*
Monthly mean maximum temperature lagged by 2 months	0.279	0.233	0.451*

* Significance (*P* < 0.05)

### Regression models

Cross-correlation analysis showed that a number of environmental and meteorological variables were significantly correlated with each other (see Additional file 1: Table S1). For instance, maximum temperature was significantly correlated with NDVI, monthly reservoir water level and reservoir water level change at each of the three dams.

Stepwise multiple regression analyses selected few environmental and meteorological variables as factors most explaining malaria incidence across the three study dams (Table 4). At the low land dam, village distance to reservoir shoreline lagged by 1 month (r^2^ = 0.468; *P* < 0.001), monthly average change in reservoir water level lagged by 2 months (r^2^ change = 0.189; *P* < 0.001) and monthly total precipitation lagged by 1 month (r^2^ change = 0.156; *P* < 0.001) together explained 81% of the monthly variability in malaria incidence. At the midland dam, village distance to reservoir shoreline lagged by 1 month (r^2^ = 0.398; *P* < 0.001), monthly reservoir water level lagged by 2 months (r^2^ change = 0.266; *P* < 0.001) and monthly total precipitation lagged by 2 months (r^2^ = 0.221; *P* < 0.001) explained 71.1% of variation in monthly malaria incidence. At the highland dam, village distance to reservoir shoreline lagged by 1 month (r^2^ = 0.324; *P* < 0.001), monthly change in reservoir water level lagged by 2 months (r^2^ change = 0.374; *P* < 0.001) and monthly mean minimum temperature lagged by 2 months (r^2^ change = 0.068; *P* < 0.001) explained 76.5% of variation in monthly malaria incidence. Overall, dam-associated factors, such as distance to shoreline or the magnitude of water level changes, were found to be the most important variables contributing to malaria incidence in nearby villages.

**Table 4:** Optimum stepwise multiple regression models relating monthly malaria incidence with environmental and climatic factors at the three dam sites

Model	Predictors	Non-standardized coefficient^a^	Adjusted R^2^	Sig.
Lowland dam				
1	Village distance from reservoir shoreline (lagged by 1 month)	− 9.46	0.468	< 0.001
2	Village distance from reservoir shoreline (lagged by 1 month), monthly change in reservoir water level (lagged by 1 month)	4..61	0.657	< 0.001
3	Village distance from reservoir shoreline (lagged by a month), monthly change in reservoir water level (lagged by 1 month), monthly total precipitation (lagged by 2 months)	2.49	0.813	< 0.001
Midland dam				
1	Village distance from reservoir shoreline (lagged by 1 month)	− 5.47	0.398	< 0.001
2	Village distance from reservoir shoreline (lagged by 1 month), monthly reservoir water level (lagged by 2 month)	3.89	0.532	< 0.001
3	Village distance from reservoir shoreline (lagged by 1 month), monthly reservoir water level (lagged by 2 months), monthly total precipitation (lagged by 2 month)	0.66	0.711	< 0.001
Highland dam				
1	Village distance from reservoir shoreline (lagged by 1 month)	− 1.98	0.324	< 0.001
2	Village distance from reservoir shoreline (lagged by 1 month), monthly change in reservoir water level	2.04	0.698	< 0.001
3	Village distance from reservoir shoreline (lagged by 1 month), monthly change in reservoir water level (lagged by 2 months), monthly minimum temperature precipitation (lagged by 1 month)	2.75	0.765	< 0.001

^a^The non-standardized coefficient of a variable is also referred as ‘effect size’ which indicates the result of a single unit increase in this variable on malaria incidence

## Discussion

This study revealed that dam-associated environmental factors and local meteorological drivers influence malaria transmission around large dams in Ethiopia. The importance of these factors, however, varied across lowland, midland and highland dam sites. Interestingly, a village’s distance from the nearest reservoir shoreline was the most important variable at all three dams, explaining 47, 40 and 32% of the monthly variation in malaria incidence at the lowland, midland and highland dams, respectively. This indicates the role that dams play in malaria transmission by providing favourable breeding habitats for malaria mosquitoes. Previous studies indicated that *An. arabiensis*, the primary malaria vector mosquito in Ethiopia, breeds along reservoir shorelines [12, 14].

Monthly reservoir water level (lagged by 2 months) was also positively correlated with monthly malaria incidence at the lowland and midland dams. This suggests that during periods of high water level, reservoir shorelines get closer to villages (as shown by distance) and contribute to increased mosquito abundance as the result of mosquito breeding in reservoir shoreline habitats. The present results also indicate that the shorter the distance between villages and reservoir shoreline the higher the malaria incidence. This is in agreement with the findings of a recent study that documented enhanced larval abundance of *An. arabiensis* and *An. pharoensis*, the major malaria vectors in Ethiopia, in lowland and midland dam areas [14]. Similar observations were also made around Lake Victoria in Kenya where the abundance of *An. gambiae* complex (of which *An. arabiensis* is a member) substantially increased during high water levels [28]. In southwest Ethiopia, Sena et al. [29] found that elevation and distance from reservoir were important factors determining malaria transmission around Gilgel-Gibe. Generally, the significant association between village distance from reservoir shoreline and malaria incidence confirms the role of dams in malaria transmission at all three dam settings.

Whilst precipitation was the most important meteorological factors associated with malaria incidence at the lowland and midland dams, minimum temperature appeared to be a significant driver of malaria incidence around the highland dam. In fact, precipitation is strongly correlated with reservoir water level as periods of high water level follow heavy rains between June and August. Teklehaimanot et al. [30] indicated that precipitation is the most important factor for malaria transmission in the lowlands of Ethiopia as mosquito breeding is largely limited by water availability. Peak malaria transmission often follows the main rainy season in Ethiopia [31]. Precipitation has a direct and indirect effect on malaria transmission around dams: it increases reservoir water level which creates potential mosquito breeding habitats along the shorelines closer to reservoir villages, and forms rain pools that mosquitoes use for breeding.

The effect of minimum temperature on malaria transmission in the highlands has long been recognized [32–35]. Temperature is a key determinant of the length of mosquito and malaria parasite life cycle [34, 36]. For instance, at 16 °C, larval development may take more than 45 days (reducing the number of mosquito generations and putting the larvae at increased risk of predators), compared to only 10 days at 30 °C [30]. However, temperature increases above 30 °C have been regarded as detrimental to parasite and mosquito development [36]. By affecting the duration of the aquatic stage of the mosquito life cycle, temperature determines the timing and abundance of mosquitoes following adequate rainfall. The feeding frequency of mosquitoes is also affected by temperature—an increase in temperature leads to increased proportions of infective mosquitoes [37]. However, the effect of temperature largely depends on elevation: as elevation increases, temperature decreases, which affects both mosquito and malaria parasite development [38]. The minimum temperature required for the development of *P. falciparum* and *P. vivax* is approximately 18 °C and 15 °C, respectively, limiting the spread of malaria at higher altitudes [39]. There is also a relationship between increasing altitude and decreasing mosquito abundance in African highlands [38]. In light of future climate change, higher temperatures could also facilitate faster desiccation of breeding habitats, compromising larval development. These effects of minimum temperature might explain the significance of this factor in determining malaria transmission rates around the highland dam in the present study.

Monthly NDVI (lagged by 1 and 2 months) was significantly correlated with malaria incidence, particularly around the lowland dam. Several studies have shown a positive significant correlation between NDVI in the preceding month and malaria in West, Central and East Africa [40–42]. However, it should be noted that temporal variation in NDVI is often highly correlated with rainfall particularly in semi-arid lowlands, as shown in the present study and others [43, 44]. In the Sudanese Savannah region of Mali, Gaudart et al. [42] reported NDVI to be an important predictor of the total surface area of breeding sites, as NDVI values increase with soil moisture. In Eritrea, Graves et al. [45] found that NDVI is a better predictor of malaria incidence than rainfall. In the absence of rainfall data, NDVI can thus be used to predict malaria risk in lowland areas.

Monthly change in reservoir water level (lagged by 2 months) was one of the most important determinants of monthly malaria incidence around the lowland and midland dams. The rate of water level change has previously been shown to determine availability of shoreline habitats for mosquito breeding around [12]. Faster water level drawdown rates, determined by the magnitude in water level change between consecutive months, were associated with low larval mosquito abundance and fewer shoreline puddles [46]. Similarly, the present study showed that a rapidly receding reservoir shoreline was associated with lower malaria incidence rates (Fig. 6). Increasing water levels, which also shorten the distance from villages to shorelines, were positively correlated with increasing malaria incidence. This also explains the seasonality of malaria around dam villages which peaks immediately after the rainy season when reservoirs fill up. Reservoir water level is thus an important factor underpinning the production of shoreline mosquito breeding habitats.

Understanding the various factors that contribute to malaria transmission is crucial in order to forecast malaria risk and devise disease control tools. Although evidence for the general impact of dams on malaria is well documented in sub-Saharan Africa [5, 6, 15], specific factors responsible for increased malaria around dams have been less clear. The present study has for the first time identified environmental and meteorological factors associated with increased malaria transmission around dams at different ecological settings. Its findings underscore the role of reservoir water levels in malaria transmission nearby, and also allow the potential of using reservoir water level management for malaria vector control to be assessed. Reservoir water level management was effectively implemented to disrupt malaria vector breeding in habitats in the Tennessee Valley, USA [47]. A recent study in Ethiopia assessed the efficacy of this approach under field experiments and found that faster drawdown rates suppress larval development [46]. However, this approach has never been applied to African dams. Future research should investigate the potential of using water level management for malaria control in existing African dams.

This study has three main limitations. First, the malaria data used were retrospective data with a 75–82% level of completeness. Active case detection would have improved confidence in the present findings relative to retrospective datasets. Second, the difference in malaria control use (e.g., bed nets) among the study dams was not considered in the modelling. Third, entomological data were not included in the modelling, although these would have contributed to the biological explanation for the lag times observed in the response of malaria infection cases to some environmental variables.

## Conclusion

Dams intensify malaria transmission in Ethiopia. The rate of reservoir water level change and village distance from reservoir shorelines were both found to be key malaria determinants around dams. As many dams are currently planned in sub-Saharan Africa, understanding the factors underlying increased malaria transmission is crucial to inform where to locate dams and communities at higher risk of the disease. Health authorities and dam operators should explore mechanisms to optimize dam operation to suppress nearby malaria transmission. Effective water level management, augmented with the existing vector control approaches, could help curb the malaria risk around large dams in Africa.

## Additional file

**Additional file 1: Table S1.** Cross-correlation of environmental and meteorological variables (values shown are r values).
