# Supplementary material for: Environmental and meteorological factors linked to malaria transmission around large dams at three ecological settings in Ethiopia
Source: Malar J. 2019 Feb 26;18:54. doi: 10.1186/s12936-019-2689-y (PMC6390543; doi:10.1186/s12936-019-2689-y)
Supplement: Supplementary file 1 — Additional file 1: Table S1. Cross-correlation of environmental and meteorological variables (values shown are r values). [file 12936_2019_2689_MOESM1_ESM.docx]

Additional file 1: Table S1. Cross-correlation of environmental and meteorological variables (values shown are r values)

| Variables | Altitude | Monthly mean minimum temperature | Monthly mean maximum temperature | Mean monthly total precipitation | Monthly average water level | Monthly change in water level | Monthly NDVI |
| --- | --- | --- | --- | --- | --- | --- | --- |
| Altitude | 1 | -0.651* | -0.432* | 0.522* | 0.334* | -0.305* | 0.682* |
| Monthly mean minimum temperature |  | 1 | 0.744^*^ | 0.206 | -0.612* | 0.101 | 0.211 |
| Monthly mean maximum temperature |  | 0.744* | 1 | 0.461* | -0.432* | 0.574* | 0.613* |
| Mean monthly total precipitation |  | 0.206 | 0.461* | 1 | 0.236 | 0.789* | 0.561* |
| Monthly average water level |  | -0.612* | -0.432* | 0.236 | 1 | 0..244 | 0.348* |
| Monthly change in water level |  | 0.125 | 0.558* | 0.789* | 0.244 | 1 | -0.455* |
| Monthly NDVI |  | 0.211 | 0.613* | 0.561* | 0.348* | -0.455* | 1 |

* Pearson correlation significant (*P* < 0.05)
